# The Influence of Climate Change on Atmospheric Deposition of Mercury in the Arctic—A Model Sensitivity Study

**DOI:** 10.3390/ijerph120911254

**Published:** 2015-09-10

**Authors:** Kaj M. Hansen, Jesper H. Christensen, Jørgen Brandt

**Affiliations:** Department of Environmental Science and Arctic Research Centre, Aarhus University, Roskilde 4000, Denmark; E-Mails: jc@envs.au.dk (J.H.C.); jbr@envs.au.dk (J.B.)

**Keywords:** mercury, climate change, Arctic, modelling, long-range transport

## Abstract

Mercury (Hg) is a global pollutant with adverse health effects on humans and wildlife. It is of special concern in the Arctic due to accumulation in the food web and exposure of the Arctic population through a rich marine diet. Climate change may alter the exposure of the Arctic population to Hg. We have investigated the effect of climate change on the atmospheric Hg transport to and deposition within the Arctic by making a sensitivity study of how the atmospheric chemistry-transport model Danish Eulerian Hemispheric Model (DEHM) reacts to climate change forcing. The total deposition of Hg to the Arctic is 18% lower in the 2090s compared to the 1990s under the applied Special Report on Emissions Scenarios (SRES-A1B) climate scenario. Asia is the major anthropogenic source area (25% of the deposition to the Arctic) followed by Europe (6%) and North America (5%), with the rest arising from the background concentration, and this is independent of the climate. DEHM predicts between a 6% increase (Status Quo scenario) and a 37% decrease (zero anthropogenic emissions scenario) in Hg deposition to the Arctic depending on the applied emission scenario, while the combined effect of future climate and emission changes results in up to 47% lower Hg deposition.

## 1. Introduction

Mercury (Hg) is a widespread pollutant that can lead to neurological and behavioural effects in infants and higher incidence of cardiovascular diseases for people with elevated exposures [1,2]. Hg exists in various forms and occurs naturally in the environment with sources from volcanic eruptions and weathering of naturally enriched rocks. The natural cycling of mercury has been perturbed by emissions from human activities, with small-scale gold mining and coal burning being the largest sources. This has led to at least a two-fold increase in atmospheric emissions since the industrial revolution in the 18th century [1]. Hg is distributed globally and is transported to the Arctic via ocean currents and rivers as well as via the atmosphere, which is assessed to be the largest transport pathway [3]. Hg is deposited in the Arctic depending on climatic parameters such as temperature, precipitation and sea-ice coverage as well as complex chemical reactions. The atmospheric transport to and deposition within the Arctic has been studied using advanced atmospheric chemistry-transport models (ACTM) [4,5,6,7,8,9]. Once at the surface, Hg enters the food webs where it bioaccumulates and biomagnifies. Levels of Hg in Arctic top predators, such as tooth whales and polar bears, exceed thresholds for adverse effects [1]. The Arctic indigenous people are exposed to high concentrations of Hg through the traditional diet rich on fish and marine mammals. Blood levels of Hg in parts of the Arctic population are higher than the safety limit [2], although declining trends are found in recent years probably associated with a change in diet [1]. High exposure is especially critical for children during development and can result in neurological disabilities such as language, learning and attention deficits [2]. There is also evidence for higher risk of cardiovascular diseases [2].

The climate has changed in recent decades with the main sign being an increase in global mean temperature. The multi-model ensembles in both the 4th and the recent 5th Intergovernmental Panel on Climate Change (IPCC) Assessment Report (AR4 and AR5) project an increase in the global mean temperature in the range of 1–6 °C by the end of the 21st century, relative to pre-industrial temperature levels [10,11]. The broad range in the projected temperature increase is linked to the various greenhouse gas emission scenarios used as input to the climate simulations. The changes are projected to be larger in the Arctic than elsewhere. The average temperature increase in the Arctic exceeds 9 °C by the end of the 21st century compared to the 1990s according to one of the intermediate emission scenarios applied in AR4, the SRES A1B scenario. The increasing temperature leads to enhanced seasonal melting of the Arctic Ocean sea ice, retreating of glaciers and melting of the Greenland ice sheet. Changes are also projected for precipitation patterns as well as in weather patterns in general.

Climate change can have an impact on the physical and chemical processes in the atmosphere, including atmospheric transport pathways, chemical composition, air-surface exchange processes, and natural emissions [12]. Changes in climate will affect the Hg exposure of humans and ecosystems within the Arctic, although the biogeochemical cycle of Hg is complex and all the effects of climate changes are not known [13].

The aim of this study is to investigate the effect of climate change on the atmospheric transport of Hg to the Arctic and the deposition within the Arctic by making a sensitivity study to see how the ACTM Danish Eulerian Hemispheric Model (DEHM) reacts to climate change forcing. We have furthermore studied the contribution from the major source areas (Asia, Europe and North America) and finally we have investigated how changes in emissions affect the deposition in the Arctic compared to changes in climate.

## 2. Experimental Section

### 2.1. Danish Eulerian Hemispheric Model (DEHM)

**Figure 1:**
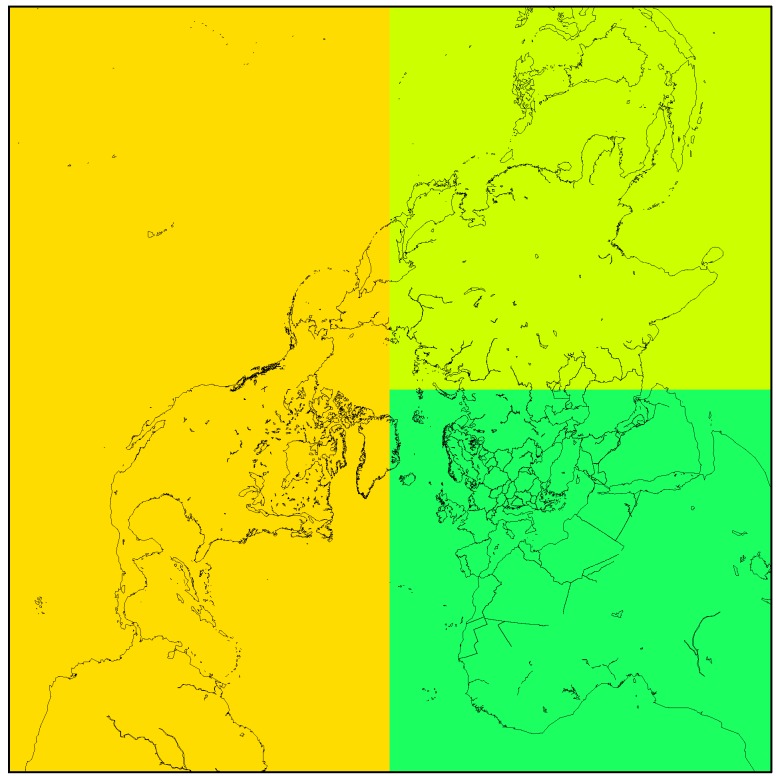
The DEHM model domain, including the sub-division into the major source areas North America (orange), Europe (dark green) and Asia (light green), applied in Section 3.2.

We applied the Danish Eulerian Hemispheric Model (DEHM) in this study, a 3D dynamic atmospheric chemistry-transport model covering the Northern Hemisphere using a polar stereographic projection (Figure 1). It has a horizontal resolution of 150 km × 150 km and 20 vertical layers extending to a height of 100 hPa. DEHM comprises a comprehensive chemistry scheme including a full SO_x_-NH_x_-NO_x_-O_3_-VOC chemistry module with 58 chemical components and 122 reactions as well as nine primary particles including black carbon [14,15,16]. In addition, there are sub-modules describing atmospheric chemistry and transport of mercury [4], fluxes and atmospheric transport of CO_2_ [17,18] as well as atmospheric transport and environmental fate of persistent organic pollutants (POPs) [19,20]. It is driven by meteorological data from a numerical weather prediction model, e.g., the MM5 weather forecast model, which is applying global analysed meteorological data as input.

The mercury chemistry scheme applied in the mercury sub-model of DEHM is based on the Tropospheric Chemistry Module (TCM) [21], which consists of three gas phase compounds: Hg^0^, HgO, HgCl_2_; 1 particulate phase Hg and nine aqueous phase compounds. Ozone is the most important oxidant for the formation of reactive gaseous mercury (RGM) and total particulate mercury (TPM) in this scheme. An additional fast oxidation rate of Hg^0^ to HgO is assumed during polar sunrise over sea-ice in the Arctic in order to simulate the Atmospheric Mercury Depletion Events (AMDEs), which accounts for up to about half of the deposition from the atmosphere [3,4,5]. It is a simple, but useful parameterization [4]. Theoretical studies suggest that reactions with atomic bromine (Br) can account for the reactivity of Hg during AMDEs [22]. The understanding of these processes is not yet complete [13], and they are not included in the model version applied in this study.

There are no re-emissions after AMDE in the Arctic in DEHM, which means that the calculated total deposition of Hg can be considered to be a model estimate of the maximum possible Hg deposition in the Arctic. Furthermore, the model does not have Hg re-emissions from ocean and soil, which are also important sources of atmospheric Hg^0^. The contributions from these sources are assumed to be included in the global background concentration of Hg^0^ of 1.5 ng/m^3^, which is used as initial and boundary conditions in all the simulations. This background concentration represents the current background concentration depending on the current anthropogenic and natural emissions as well as re-emissions. However, it is possible to estimate the influence of the anthropogenic emissions on the background concentration via post processing if the contribution from the global background concentration is known. This is done by assuming that only anthropogenic emissions of Hg^0^ contribute to the global background concentration and that the natural emissions and re-emissions are assumed to be constant with 4200 tons Hg based on estimates from AMAP [1]. The background concentration can then be calculated from the following expression:

(1) $$Hg_{back}^{0}=1.5ng/m^{3}\frac{\left(Hg_{used\;emis}^{0}+4200\;tonnes\right)}{\left(Hg_{emis\;2005}^{0}+4200\;tonnes\right)}$$

In all the simulations presented here, the contribution from the background concentration is known. Therefore, this post-processing procedure has been used for all the presented results. The ozone concentrations are provided by the SOx-NHx-NOx-O_3_-VOC chemistry module, and Black Carbon concentrations, which are applied in the model for the production of particulate Hg in the aqueous phase chemistry, are provided by the particle module in DEHM.

Model evaluations against measured Hg concentrations in air show reasonable agreement with predicted concentrations from DEHM with input of real meteorology [4,5,6]. DEHM has also successfully been applied to study the transport and fate of a range of other chemical compounds to the Arctic, including sulphur and sulphate [15], Pb [6], Persistent Organic Pollutants (POPs) [19,20,23] and Decamethylcyclopentasiloxane [24]. The model was previously applied to study the effect of climate change on future atmospheric levels of ozone and particulate matter [25,26,27,28,29] and related impacts on health [30] as well as on the fate of POPs in the Arctic [31].

### 2.2. Model Set-Up

We have performed two model experiments in this study. The first model experiment was made to estimate the influence of climate change on the atmospheric transport of Hg to the Arctic and the deposition within the Arctic. This experiment was also applied to identify the major source areas of Hg to the Arctic and to study if there is a change in the importance of the different source areas in the applied climate change scenario. The second model experiment was made to estimate the effects of changes in emissions compared to the effect of changes in climate conditions. For the first experiment we made four sets of simulations, one with all sources, one with no sources in North America (NA), one with no sources in Europe (EU), and one with no sources in Asia (AS), see Figure 1 for the subdivision. By using these model simulations, it is possible to estimate the contribution from the different source areas including the boundary and initial conditions of Hg^0^, *i.e*., the contribution from the global background concentration using a simple subtraction method since all the Hg related processes in the model are linear. In these simulations, we applied the most recent global Mercury emission inventory for 2005 [32].

For the second model experiment, we performed five sets of simulations with different future emission scenarios for the year 2020 [32], one assuming status quo emissions (SQ), one with extended emissions control (EXEC), one assuming the maximum feasible technological reduction (MFTR) and one without anthropogenic emissions (zero emission).

Each set of simulations in both model experiments consists of two ten-year time slices representing present (1990–2000) and future (2090–2100) climates. A 30-year period is usually applied as a climate normal in climate studies, however, the ten-year time slices were chosen in this study to reduce the computation time and this approach has proven to be successful in several studies [25,26,27,28,31]. We have applied the same emissions to the atmosphere in each of the sets of simulations. With this set-up, the difference in predicted concentrations and depositions for each set of simulations arises only from the effect the difference in climate input has on the transport and deposition processes, and it is possible to investigate the response of DEHM to a changed climate forcing.

To drive the simulations, we have applied meteorological data from a model simulation made with the ECHAM5/MPI-OM model [33,34,35] simulating the SRES A1B scenario [36]. The SRES A1B scenario projects the global average temperature to increase by ~3 °C by the end of the 21st century with large seasonal and regional differences in the warming. The average sea ice extent in the Arctic is estimated to retreat by approximately 40%, and over the Barents Sea, the sea ice is predicted to vanish completely by the end of the 21st century. The globally averaged precipitation changes only slightly, although there are large regional and seasonal differences. The winter precipitation over the temperate and arctic regions is projected to increase by 10%–50%. The global temperature change related to the SRES A1B scenario from AR4 is quite similar to the RCP6 scenario used in the IPCC AR5 [11]. The SRES A1B is only one of several climate change scenarios and represents an intermediate development of the climate. This is a sensitivity analysis to investigate the response of the model system to changed climate input. One thing that should be kept in mind with the interpretation of such sensitivity analyses is that the DEHM model, as many other models, has parameterizations of processes which are tuned to the current climate in order to improve the present day model performance. The processes may change with climate conditions and the model may thus not have a reliable response to a changed climate input.

## 3. Results and Discussion

### 3.1. The Effect of Climate Change on Deposition in the Arctic

**Figure 2:**
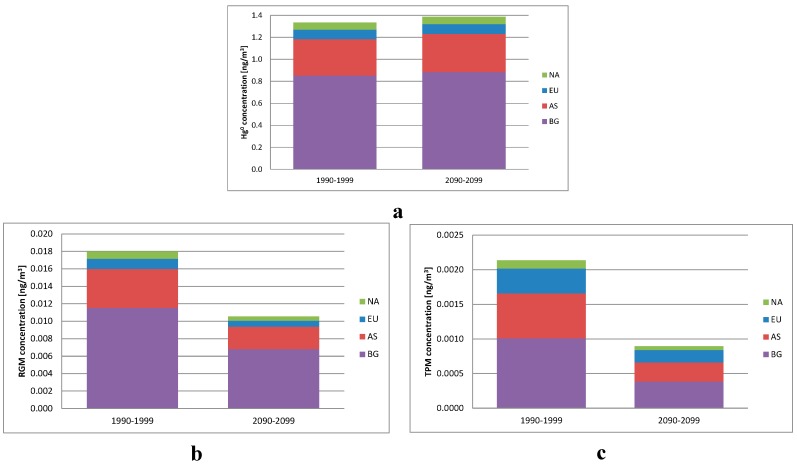
The average Artic air concentrations of Hg^0^ (**a**), Reactive Gaseous Mercury (RGM = HgO + HgCl_2_) (**b**) and Total Particular Mercury (TPM = primary + oxidized particulate Hg) (**c**) for the 1990s and 2090s divided into the contribution from North America (NA), Europe (EU), Asia (AS), and background concentration (BG).

The average Arctic air concentrations of Hg^0^, Reactive Gaseous Mercury (RGM = HgO + HgCl_2_) and Total Particular Mercury (TPM = primary + oxidized particulate Hg) are shown in Figure 2 for the 1990s and the 2090s. The average concentration of Hg^0^ is 4% higher in the 2090s compared to the 1990s, while the concentrations of both RGM and TPM are lower with 41% and 62%, respectively.

The main reason for the differences between the two decades in the model is that the parameterization of the fast oxidation of Hg^0^ to HgO during the AMDEs depends on the sea-ice fraction. The lower sea-ice fraction in the 2090s compared to the 1990s will result in a lower oxidation of Hg^0^ to HgO and therefore a higher concentration of Hg^0^ and lower concentrations of RGM and TPM.

**Figure 3:**
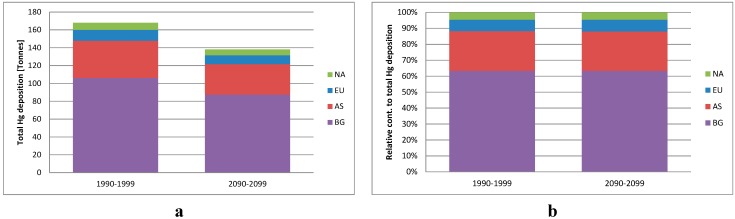
The total deposition of Hg in tons/year with the contribution from the different source areas including indirect contribution (**a**) and the relative contribution from the different source areas to the total deposition of Hg for the Arctic (**b**) for the two studied decades.

In Figure 3, the total annual deposition of Hg north of the Arctic Circle (66.5^o^ N) is shown. The total deposition is 18% lower in the 2090s than in the 1990s, and the difference is statistically significant. RGM is the major component of the deposition due to a high dry deposition velocity to the surface, and the lower RGM air concentrations in the 2090s result in lower depositions.

**Figure 4:**
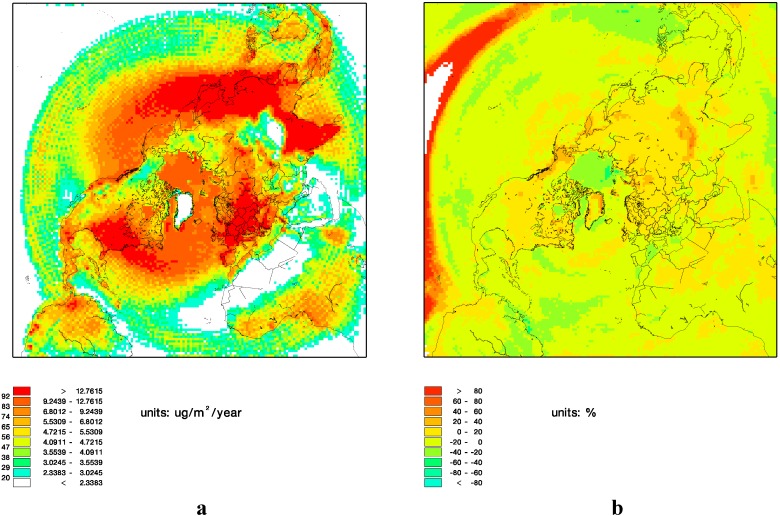
Average of the total annual mercury deposition for the 1990s (**a**) and change in deposition in percent from the 1990s to the 2090s (**b**).

The spatial distribution of the average total annual deposition in the 1990s is shown in Figure 4 together with the difference in percent between the average total annual deposition in the 1990s and the 2090s. The deposition to the continents is significantly higher in the 2090s than in the 1990s due to significantly higher ozone concentrations in the troposphere, which is one of the main oxidant paths of mercury in the DEHM model. The deposition to the Arctic Ocean is significantly lower in the 2090s than in the 1990s due the reduction in sea ice coverage.

There are several processes not accounted for in the DEHM model that potentially can affect the estimate of the importance of climate change for the Hg deposition within the Arctic. One process is re-volatilization from the surface. This is partly accounted for by the background concentration as described in section 2.1. It has been speculated that a reduction in sea-ice cover will lead to an enhanced re-volatilization from the Arctic Ocean, while another effect is that TPM deposited on the ice free water is less likely to re-volatilize immediately than when deposited on sea ice and snow and the combination of these two effects may be nearly neutral [13]. On the other hand, the volatilization is dependent on the gradient between the concentrations in air and water, and a higher concentration of Hg^0^ in air in the 2090s combined with a larger ice-free part of the Arctic Ocean will lead to a lower re-volatilization of Hg^0^ from the surface. If re-volatilization was accounted for in the DEHM model this would have reduced the Hg^0^ concentrations in air (corresponding to a lower background concentration in the model), which would lead to lower estimated depositions to the surface. However, we estimate that this effect on the reduced oxidation to RGM and subsequent lower deposition is negligible compared to the effect of changes in sea-ice coverage.

Theoretical studies [22] and combined experiments and model studies [5] indicate that reactions of Hg with atomic bromine can account for the conversion of Hg^0^ to RGM during AMDEs, although the understanding of these processes is not yet complete [13]. It has been suggested that higher temperatures will slow down the gas-phase oxidations and thus reduce the depositions if the present Br levels are unchanged [13]. There are uncertainties regarding the origin of atmospheric Br that are associated with snow, refreezing leads and formation of first-year sea-ice [13,37]. Following the increasing atmospheric temperature the formation of first year sea ice is expected to increase, which could lead to an increase in release of Br to the atmosphere, which again will result in an increase of the rate of Hg deposition during AMDEs [13].

### 3.2. The Contribution from Major Source Areas

The contribution from the four different source areas: North America (NA), Europe (EU), Asia (AS), and the background (BG) is also shown in Figure 2. For both Hg^0^ and RGM, the contribution from the global background is 64% of the total concentration in the 1990s, while Asia contributes with 25%, Europe with 6% and 5% arrives from North America. The difference in the contributions for the two decades is less than 1% for Hg^0^ and RGM, which means that the relative contribution from the source areas does not depend on the climate according to these model simulations. For particulate Hg, the contribution from the background is smaller (47% for the 1990s, 43% for the 2090s), mainly because of the transport of primary particulate Hg. Europe contributes with 17% in the 1990s and 20% in the 2090s, Asia with 30% and 31% for the two decades and North America with 6% for both decades. This indicates that the transport of primary particulate Hg, especially from Europe, is enhanced in the 2090s compared to the 1990s under the applied climate change scenario. Results from the DEHM model also indicates that the atmospheric transport of POPs to the Arctic is larger in a future warmer climate [31].

**Figure 5:**
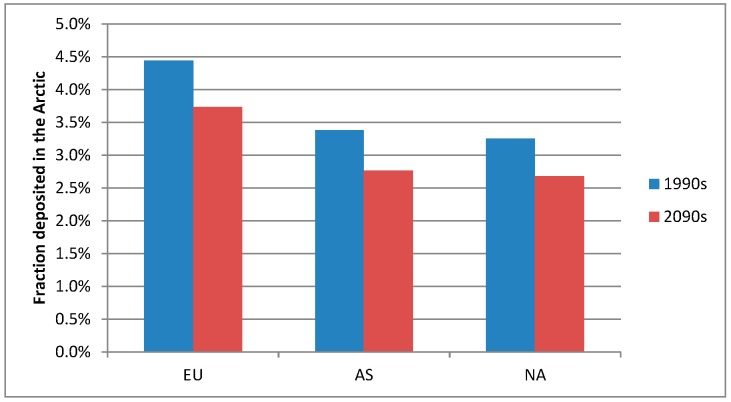
The fraction of the total emitted Hg deposited in the Arctic originating from each of the three source areas: Europe (EU), Asia (AS), and North America (NA) in the 1990s (Blue) and in the 2090s (red).

The relative contribution to the deposition is also shown in Figure 3 and these are quite similar to the relative contributions of Hg^0^ concentrations as described above, reflecting that Hg is mainly transported to the Arctic as Hg^0^ which is oxidized to RGM and particulate Hg during the transport and some of this oxidized Hg is deposited. The larger atmospheric transport of TPM in the 2090s than in the 1990s is so small compared to the Hg^0^ that it does not influence the overall relative contribution distribution. The global background is the most important contributor. There are only very small differences (<0.2%) in the relative contributions for the two decades indicating that climate change do not have a large influence on the relative contribution of the source areas. All the source areas in the Northern Hemisphere contributes to a large background pool of Hg^0^ and this is getting well mixed before the Hg is deposited because of the long lifetime of Hg^0^ in the atmosphere (1 year). However, there are some differences between the source areas in how large a fraction of the total emission that is deposited in the Arctic (Figure 5). Europe is relatively the most important source area from which around 4.4% of the total Hg emission is deposited in the Arctic in the 1990s, while it is 3.3% for Asia and 3.2% for North America. This shows that the emissions from Europe are more efficiently transported to the Arctic compared to emissions from Asia and North America, which are both located more southerly and in addition have a longer transport pathway before they arrive in the Arctic. Common for all three source areas is that these fractions are lower in the 2090s compared to the 1990s (3.7%, 2.7% and 2.6% for EU, AS, and NA, respectively), corresponding to 16%–18% lower. This is due to the lower deposition owing to the lower sea-ice fraction in the 2090s than in the 1990s.

Dastoor *et al*. [9] investigated the major source areas for Hg deposition to the Canadian Arctic using the Global/Regional Atmospheric Heavy Metals Model (GRAHM). Although their division of source areas was finer than the one applied in this study and their model domain did not cover the most eastern part of the Arctic, some similarities are seen. The contribution from re-emission and natural sources (corresponding to the background in this study) accounts for an estimated two thirds of the deposition to the Canadian Arctic, while (East) Asia is the major anthropogenic source area followed by Europe and North America.

### 3.3. The Contribution from Emission Changes vs. Climate Changes

**Figure 6:**
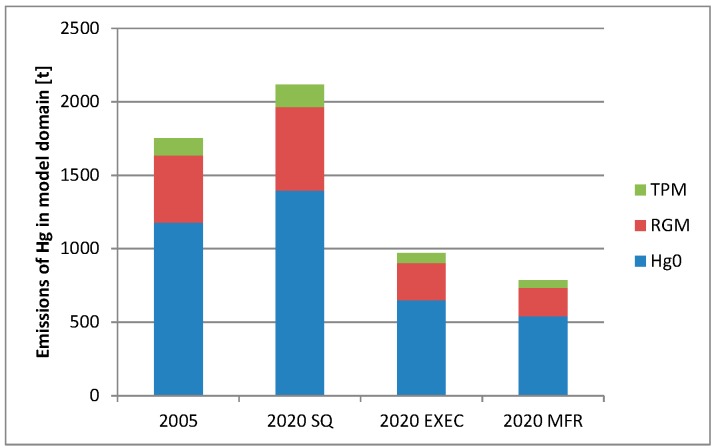
The total annual emission in tons Hg within the model domain for the four different emission scenarios, split up in emission of Hg^0^, reactive Hg (RGM) and particulate Hg (TPM).

To study the effect of emission changes *versus* the effect of climate changes, three different emission scenarios were used for the target year of 2020: the “Status Quo” (SQ) scenario, the “Extended Emissions Control” (EXEC) scenario, and the “Maximum Feasible Technological Reduction” (MFTR) scenario [32]. Detailed emission scenarios for a more distant future (e.g., 2090s) do not exist. In Figure 6 the emissions of Hg^0^, reactive Hg (RGM), and particulate Hg (TPM) are shown. The total emissions inside the model domain for the SQ scenario is 21% higher than the 2005 emissions, while the EXEC and MFTR are 45% and 55% lower, respectively.

**Figure 7:**
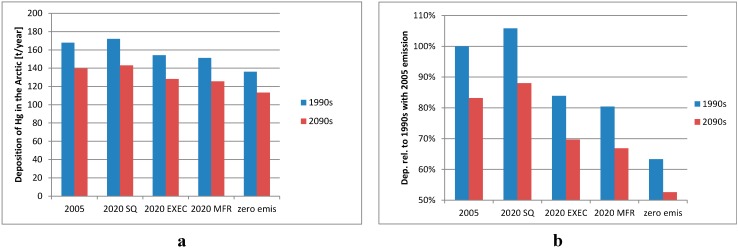
Total mercury deposition in Tons Hg/year to the Arctic for all different emissions scenarios from DEHM (**a**) and the relative difference in % (**b**) for the 1990s (blue) and the 2090s (red).

We have performed five sets of simulations. The first set of simulations is with the global Mercury inventory for 2005, three sets of simulations were with each of the 2020 emission scenarios (SQ, EXEC and MFTR), and the last set was with no anthropogenic emissions, called “zero emission”. In Figure 7, the total deposition to the Arctic (a) and the relative changes compared to the 2005 basic simulation for the 1990s (b) are shown for all 10 model simulations. The MFTR results in 20% lower, the EXEC in 16% lower, while the SQ scenario results in a 6% higher total deposition in the 1990s compared to the 2005 basic emissions simulation. If the anthropogenic emissions are removed the deposition to the Arctic will be 37% lower for the 1990s. The decrease in depositions is in excellent accordance with previous model estimates using the same emission scenarios, with predictions of an increase in deposition in the Canadian part of the Arctic of approximately 5% for the SQ emission scenario and decreases in deposition of 15%–17% for the EXEC and 18%–20% for the MFTR emission scenarios [9].

**Figure 8:**
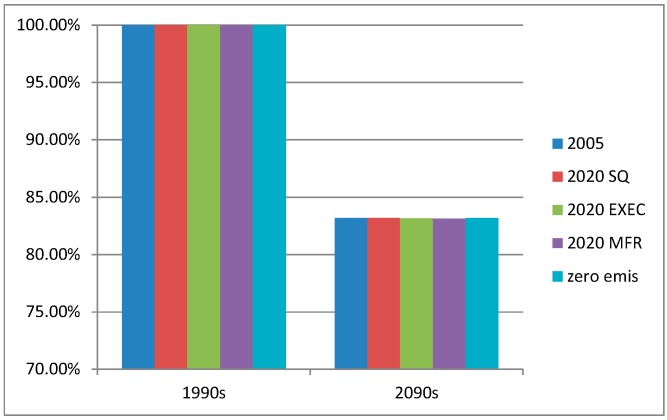
The relative changes of the total mercury deposition to the Arctic areas north of Polar Circle for all emissions scenarios between the 1990s and the 2090s.

The relative change in Hg deposition to the Arctic between the 1990s and the 2090s is 18% for all five emissions scenarios (Figure 8). The changes in deposition for the future climate scenario are thus not influenced by the differences in emissions just as for the major source areas.

According to the model simulations presented here, the decrease of the Hg deposition due to climate changes are smaller than the decrease due to emission changes in both the MFTR and zero emission scenarios; 18% *vs*. 20% or 37%, respectively. When combining the MFTR and zero emission scenarios with changed climate input, the model system predicts total Arctic Hg deposition that are 33% lower for the MFTR emission scenario and 47% lower for zero emission scenario for the end of 21st century compared to end of 20th century (Figure 7). The combined effect of changing both emissions and climate input is different than the sum of the simulation with changed emission and the simulation with changed climate.

The change of Hg deposition in Arctic due to emission change over time should be even larger, because the amount of global biologically available Hg in soil and ocean will change slowly due to the changed atmospheric fluxes and the removal of biologically available Hg, e.g., by transport of Hg to the deep ocean or by sedimentation, and this will change the remissions and the global atmospheric background further.

## 4. Concluding Remarks

We have performed a series of model simulations to test how the Danish Eulerian Hemispheric Model (DEHM) reacts to climate change forcing. We have investigated how the atmospheric concentrations and depositions of Hg in the Arctic changes under a moderate climate change scenario. We have furthermore studied the contribution from the major source areas (Asia, Europe and North America) and finally we have investigated how changes in emissions affect the deposition in the Arctic compared to changes in climate.

With the applied SRES A1B climate change scenario, the DEHM model predicts that Hg^0^ air concentrations is 4% higher, while RGM and TPM are 41% and 62% lower by the end of the 21st century compared to the end of the 20th century. This results in 18% lower Hg deposition to the Arctic. The reason for these differences is the smaller sea-ice coverage predicted in the 2090s than in the 1990s. This results in a lower oxidation of Hg^0^ to HgO and therefore a higher concentration of Hg^0^ and lower concentrations of RGM and TPM and thus lower deposition.

We can see from the investigation of the major anthropogenic source areas that Asia contributes with 25% of the deposition to the Arctic, followed by Europe (6%) and North America (5%), with the rest arising from the background concentration. The fraction of contribution does not change significantly under the applied climate change scenario.

DEHM predicts between a 6% increase (Status Quo scenario) and a 37% decrease (zero anthropogenic emissions scenario) in Hg deposition to the Arctic under present day climate depending on which of the four emissions scenarios that are applied. The relative change in Hg deposition to the Arctic between the 1990s and the 2090s is the same for all applied emissions scenarios.

The model system predicts total Arctic Hg deposition that are up to 47% lower for the end of 21st century compared to end of 20th century when combining the emission change scenarios with changed climate input. The combined effect of changing both emissions and climate input is different than the sum of the simulation with changed emission and the simulation with changed climate.

This study indicates that the deposition of Hg to the Arctic decreases in a future warmer climate, although the changes are moderate. Furthermore, future climate mitigation measures are expected to reduce the atmospheric emissions of Hg further [2], which will also reduce the deposition. Lower atmospheric depositions in the future would lead to a lower uptake of Hg in the food webs and hence a lower exposure of the Arctic population to Hg if there were no other changes. However, climate change may alter other factors that influence the exposure, such as the air-surface exchange flux and release of Hg from reservoirs stored in glaciers and thawing permafrost, the inflow of riverine Hg to the Arctic Ocean, the methylation/demethylation in the Arctic Ocean and the fresh water systems, as well as the structure and dynamics of fresh water and marine food webs [1,13]. The results generated in this study indicate that climate change will reduce Hg depositions to the Arctic, although emission reductions appear to have a larger effect. This knowledge is valuable for decision makers when developing future emission reduction policies to protect the vulnerable ecosystems in the Arctic. However, the results are not very strong to form policies on alone due to the uncertainties. They could be applied in a broader understanding of the effects of Hg exposure to the Arctic population combining them with knowledge on bioaccumulation, human effects, and societal changes (e.g., diet) in a cross disciplinary study to form an integrated model system describing the cycling of Hg from source trough atmospheric transport, bioaccumulation in the food webs, exposure to top predators and humans and the caused effects.

Only one climate change scenario has been applied in this study, and the predicted changes in the deposition of Hg due to changes in climate input is a sensitivity study of the DEHM model system and this does not necessarily reflect a correct climate response in the real atmosphere. There are uncertainties in the applied parameterizations both in this and in other models due to the knowledge gaps in the biogeochemical cycle of Hg [13]. A slowly increasing trend in modelled atmospheric depositions in the Canadian Arctic in the period 1990–2005 is associated with changes in climate parameters [9], and this indicates an opposite effect of climate change on deposition to the predictions from this study. Further studies using measurements, other models and other climate change and emission change scenarios are thus needed to resolve this matter. There is also a need for the scientific community to further study the physical and chemical processes that are the most uncertain and that could become more important in a future warmer climate, notably the influence of bromine chemistry on the Hg deposition as discussed in this study. Combining measurements and models can improve our understanding of these processes.
